# CpaA Is a Glycan-Specific Adamalysin-like Protease Secreted by Acinetobacter baumannii That Inactivates Coagulation Factor XII

**DOI:** 10.1128/mBio.01606-18

**Published:** 2018-12-18

**Authors:** Ursula Waack, Mark Warnock, Andrew Yee, Zachary Huttinger, Sara Smith, Ayush Kumar, Alban Deroux, David Ginsburg, Harry L. T. Mobley, Daniel A. Lawrence, Maria Sandkvist

**Affiliations:** a Department of Microbiology and Immunology, University of Michigan Medical School, Ann Arbor, Michigan, USA; b Department of Internal Medicine, Division of Cardiovascular Medicine, University of Michigan Medical School, Ann Arbor, Michigan, USA; c Life Sciences Institute, University of Michigan, Ann Arbor, Michigan, USA; d Department of Microbiology, University of Manitoba, Winnipeg, Manitoba, Canada; e Grenoble Alpes University Hospital, Grenoble, France; f Howard Hughes Medical Institute, Ann Arbor, Michigan, USA; g Department of Internal Medicine, University of Michigan Medical School, Ann Arbor, Michigan, USA; h Department of Human Genetics, University of Michigan Medical School, Ann Arbor, Michigan, USA; i Department of Pediatrics, University of Michigan Medical School, Ann Arbor, Michigan, USA; University of Washington

**Keywords:** *Acinetobacter baumannii*, CpaA, factor XII, O-linked glycosylation, metalloprotease, thrombosis, type II secretion system

## Abstract

Antibiotic-resistant Acinetobacter baumannii is increasingly recognized as a cause of difficult-to-treat nosocomial infections, including pneumonia, wound infections, and bacteremia. Previous studies have demonstrated that the metalloprotease CpaA contributes to virulence and prolongs clotting time when added to human plasma as measured by the activated partial thromboplastin time (aPTT) assay. Here, we show that CpaA interferes with the intrinsic coagulation pathway, also called the contact activation system, in human as well as murine plasma, but has no discernible effect on the extrinsic pathway. By utilizing a modified aPTT assay, we demonstrate that coagulation factor XII (fXII) is a target of CpaA. In addition, we map the cleavage by CpaA to two positions, 279-280 and 308-309, within the highly glycosylated proline-rich region of human fXII, and show that cleavage at the 308-309 site is responsible for inactivation of fXII. At both sites, cleavage occurs between proline and an O-linked glycosylated threonine, and deglycosylation of fXII prevents cleavage by CpaA. Consistent with this, mutant fXII (fXII-Thr309Lys) from patients with hereditary angioedema type III (HAEIII) is protected from CpaA inactivation. This raises the possibility that individuals with HAEIII who harbor this mutation may be partially protected from A. baumannii infection if CpaA contributes to human disease. By inactivating fXII, CpaA may attenuate important antimicrobial defense mechanisms such as intravascular thrombus formation, thus allowing A. baumannii to disseminate.

## Abstract (precis)

Ventilator-associated pneumonia and catheter-related bacteremia are the most common and severe infections caused by Acinetobacter baumannii. Besides the capsule, lipopolysaccharides, and the outer membrane porin OmpA, little is known about the contribution of secreted proteins to A. baumannii survival *in vivo*. Here we focus on CpaA, a potentially recently acquired virulence factor that inhibits blood coagulation *in vitro*. We identify coagulation factor XII as a target of CpaA, map the cleavage sites, and show that glycosylation is a prerequisite for CpaA-mediated inactivation of factor XII. We propose adding CpaA to a small, but growing list of bacterial proteases that are specific for highly glycosylated components of the host defense system.

## INTRODUCTION

Antibiotic resistance in community and health care settings is increasingly common and is especially alarming for Gram-negative pathogens as they are rapidly becoming resistant to the majority of available drugs (1). Among the more serious health care-associated Gram-negative infections are those caused by antibiotic-resistant Acinetobacter baumannii (http://www.cdc.gov/drugresistance). While rarely acquired in the community, the recently emerging pathogen A. baumannii survives remarkably well in the hospital environment and adapts easily in individuals with compromised host defenses where it is capable of causing pneumonia, wound and urinary tract infections, bacteremia, and meningitis (2). The escalating prevalence of life-threatening infections caused by multidrug-resistant strains of A. baumannii highlights the urgent need for new therapeutic interventions. Analysis of mutants with single gene deletions or transposon insertions in different infection models has identified a number of A. baumannii factors important for *in vivo* persistence that may be suitable for therapeutic targeting (3 – 9). For example, survival in the bloodstream and subsequent colonization by A. baumannii in the spleen and liver is supported in part by the type II secretion system (T2SS) (8), a multiprotein complex that transports a variety of enzymes, including lipases and proteases, across the outer membranes of *Acinetobacter* and other bacterial pathogens (8, 10 – 13). This was demonstrated using a neutropenic murine model for bacteremia, in which the T2SS mutant Δ*gspD* strain was outcompeted by the wild-type (WT) A. baumannii strain. Similar colonization defects were observed with other *gspD* mutants of A. baumannii and the closely related Acinetobacter nosocomialis in murine pulmonary infection models (14, 15). LipA, a lipase that is secreted by the T2SS and required for lipid utilization, contributes to the *in vivo* colonization of A. baumannii (8). However, the Δ*lipA* mutant does not show the same colonization deficiency as the Δ*gspD* mutant in the bacteremic murine model, suggesting that additional proteins delivered by the T2SS have a positive influence on the survival of A. baumannii in the mammalian host.

Recently, the metalloprotease CpaA was shown to be secreted by the T2SS in A. nosocomialis (14). In addition to a functional T2SS, it was demonstrated that CpaA folding and/or secretion also requires CpaB, a putative chaperone (16). Using a murine pneumonia model, the latter study also indicated that CpaA aids in A. nosocomialis dissemination. CpaA is also expressed by several A. baumannii isolates, including AB031, and has been suggested to interfere with blood coagulation, as cell-free culture supernatant from AB031, but not that of a *cpaA* deletion mutant, increases the clotting time of normal (healthy) human plasma in an activated partial thromboplastin time (aPTT) assay (17). The aPTT assay primarily measures the activity of the contact activation and common pathways of coagulation and is routinely used in combination with the prothrombin time (PT) assay for the screening of coagulation factor deficiencies.

The contact activation (or intrinsic) pathway is initiated when circulating coagulation factor XII (fXII) (Hageman factor) comes in contact with negatively charged artificial or biological surfaces such as polyphosphates, nucleic acids, phospholipids, misfolded proteins, or lipopolysaccharides (LPS) resulting in its autoactivation (18). Active fXII (fXIIa), in turn, cleaves and activates factor XI. In a subsequent cascade of proteolytic events, which generates thrombin and induces fibrin polymerization, a clot is ultimately formed (see Fig. S1 in the supplemental material) (19). FXIIa also activates prekallikrein (PK) to generate kallikrein, which in reciprocal fashion produces more fXIIa through a feedback loop. In addition, kallikrein targets high-molecular-weight kininogen to release bradykinin, a proinflammatory mediator (20, 21) and triggers activation of the classic complement pathway (21). A recent study has also identified a direct role of autocrine fXII in upregulation of neutrophil function that promotes neutrophil trafficking (22).

10.1128/mBio.01606-18.1 FIG S1 Schematic of the contact activation (intrinsic) pathway of coagulation. f, coagulation factor. Download FIG S1, PDF file, 0.1 MB. Copyright © 2018 Waack et al. 2018 Waack et al. This content is distributed under the terms of the Creative Commons Attribution 4.0 International license.

Here, we demonstrate that CpaA increases the clotting time in the aPTT assay through proteolytic cleavage and inactivation of fXII in both human and murine plasma. Specifically, CpaA targets the glycosylated, proline-rich domain of fXII. Deglycosylation of fXII and substitution of one of the glycosylated amino acids in the proline-rich region of fXII reduce the cleavage efficiency of CpaA.

## RESULTS

### CpaA interferes with the contact activation pathway in human plasma.

**FIG 1:**
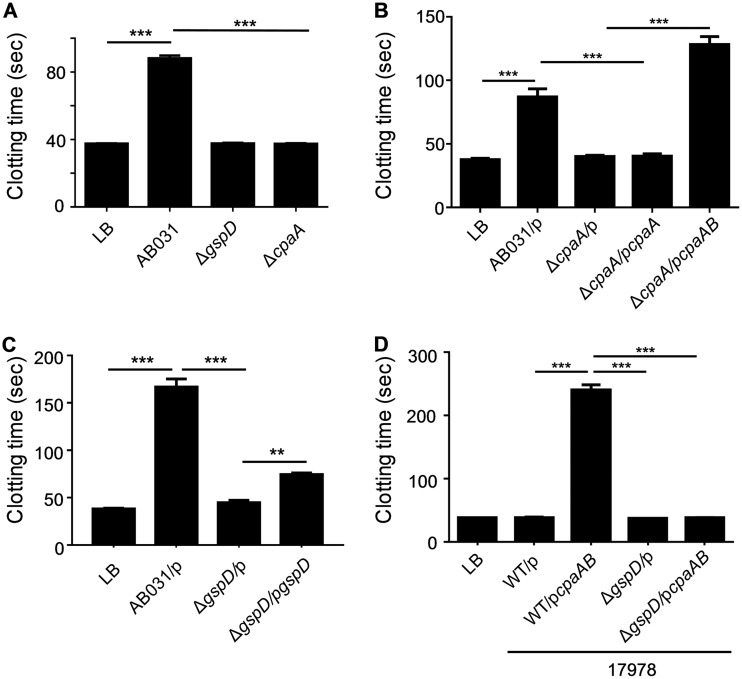
Clotting time of human plasma is increased when incubated with culture supernatant from A. baumannii but not from strains deficient in either CpaA or the T2SS. An aPTT assay was performed after incubation of normal human plasma with culture supernatant from A. baumannii strain AB031, AB031 Δ*cpaA*, or AB031 Δ*gspD* (A), AB031 or Δ*cpaA* with either empty vector pMMB960 (p), p*cpaA* or p*cpaAB* (B), AB031 or Δ*gspD* with either empty vector pMMB67 (p) or p*gspD* (C), and A. baumannii strain 17978 or the T2SS mutant 17978 Δ*gspD* with either empty vector pMMB67 (p) or p*cpaAB* (D). The growth medium LB served as a negative control. Values that are significantly different are indicated by ordinary one-way ANOVA (*n* = 6) are indicated by bars and asterisks as follows: ***, *P* ≤ 0.001; **, *P* ≤ 0.01.

CpaA, a member of the adamalysin family of secreted metalloproteases, has been found to inhibit clotting in human plasma using the aPTT assay (17). We confirmed that culture supernatant from the WT AB031 strain, but not from the isogenic Δ*cpaA* mutant, increases the clotting time in an aPTT assay in human plasma (Fig. 1A). Complementation of the Δ*cpaA* mutant required coexpression of both *cpaA* and *cpaB*, the gene coding for a putative chaperone, as no complementation was observed with *cpaA* alone (Fig. 1B). This is consistent with the results of a recent study that used both *cpaA* and *cpaB* to restore secretion of CpaA in an in-frame, unmarked *cpaA* deletion mutant of A. nosocomialis and may be due to the presence of a cryptic *cpaB* promoter within *cpaA* or to differential folding of the transcript, resulting in reduced levels of expression of *cpaB* in the absence of *cpaA* (16). Similarly to the Δ*cpaA* mutant, culture supernatant from the T2SS mutant Δ*gspD* strain was lacking CpaA (Fig. S2A and B) and had no effect on clotting (Fig. 1A), and while full complementation was not obtained with plasmid-encoded *gspD* due to only partial restoration of CpaA secretion (Fig. S2C), the increase in clotting time in the presence of p*gspD* compared to the clotting time of the empty vector control was statistically significant (Fig. 1C). Lack of full complementation has been reported previously for some T2SS mutants, including *gpsD* mutants and can be due to expression levels that are either too low or too high that may interfere with the stoichiometry and thus, the function of the T2SS (15, 23 – 26). Cloning and overexpression of *cpaA* and the putative chaperone-encoding *cpaB* also resulted in extracellular release of CpaA in a T2SS-dependent fashion in the reference strain ATCC 17978, which does not encode its own *cpaA* and *cpaB* genes (Fig. S3A). Not surprisingly, culture supernatant from WT ATCC 17978 overexpressing the protease also extended the clotting time in the aPTT assay, while expression of plasmid-encoded *cpaA* and *cpaB* in the 17978Δ*gspD* mutant did not diminish the clotting function (Fig. 1D). The effect on the clotting time in the aPTT assay was dose dependent (Fig. S3B), and the CpaA activity was significantly higher in the culture supernatant of the ATCC 17978 strain than in the original AB031 strain due to overexpression. This material, in which the concentration of CpaA was approximately 0.4 mg/ml, was used in all subsequent analyses to characterize the activity and specificity of CpaA. While we confirmed that CpaA does inhibit clotting in human plasma in the aPTT assay, unlike Tilley et al. (17), we found no significant effect of CpaA in a PT assay (Fig. S4A), which measures the activity of the extrinsic pathway. Despite not having an effect in the PT assay, CpaA does cleave coagulation factor V (fV) (Fig. S4B), but this cleavage neither inactivates nor activates fV. These findings led us to focus on the intrinsic pathway and the aPTT assay.

10.1128/mBio.01606-18.2 FIG S2 Secretion of CpaA is dependent on the A. baumannii T2SS. (A) Concentrated culture supernatants from strain AB031 and the T2SS mutant Δ*gspD* strain were analyzed by SDS-PAGE and Coomassie staining. The AB031 Δ*cpaA* mutant served as a negative control. (B) CpaA was excised from the gel and subjected to LC-MS/MS analysis, which identified 45 exclusive unique peptides, 121 exclusive unique spectra, and a total of 448 spectra. The resulting sequence coverage (highlighted in yellow) was 84%. No peptide representing the signal peptide was identified, as the signal peptide is removed during transport of CpaA across the cytoplasmic membrane. (C) Concentrated culture supernatants from AB031 and the T2SS mutant Δ*gspD* with empty vector pMMB67 (p) or plasmid-encoded *gspD* (p*gspD*) were analyzed by SDS-PAGE and Coomassie staining. Download FIG S2, PDF file, 0.2 MB. Copyright © 2018 Waack et al. 2018 Waack et al. This content is distributed under the terms of the Creative Commons Attribution 4.0 International license.

10.1128/mBio.01606-18.3 FIG S3 Overexpression, secretion, and activity of CpaA in the ATCC 17978 strain. (A) Concentrated culture supernatants from ATCC 17978 or the Δ*gspD* mutant containing empty vector (p) or a plasmid carrying the *cpaA* and *cpaB* genes (p*cpaAB*) were analyzed by SDS-PAGE and Coomassie staining. (B) Increasing amounts of culture supernatant from ATCC 17978/p*cpaAB* were added to normal human plasma (NHP), and an aPTT assay was performed; *n* = 6. Download FIG S3, PDF file, 0.1 MB. Copyright © 2018 Waack et al. 2018 Waack et al. This content is distributed under the terms of the Creative Commons Attribution 4.0 International license.

10.1128/mBio.01606-18.4 FIG S4 CpaA has no effect on clotting in a PT assay. (A) Increasing amounts of supernatant from ATCC 17978/p*cpaAB* containing CpaA was added to NHP and analyzed for clotting using a PT assay; *n* = 6, ***, *P* < 0.001. The snake venom protease RVV-V, a factor V activator, and the buffer TBS were included as controls. (B) NHP was incubated with or without CpaA and subjected to SDS-PAGE and immunoblotting using anti-fV antibody. fV-deficient plasma incubated in the absence of CpaA was used as a negative control. A representative blot is shown. Download FIG S4, PDF file, 0.2 MB. Copyright © 2018 Waack et al. 2018 Waack et al. This content is distributed under the terms of the Creative Commons Attribution 4.0 International license.

### CpaA targets factor XII.

**FIG 2:**
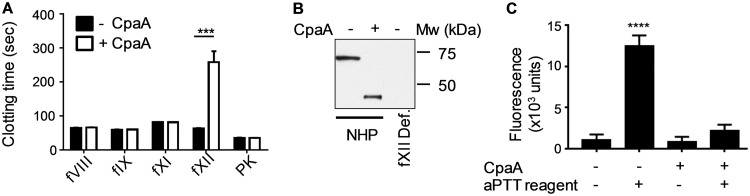
CpaA cleaves fXII. (A) Normal human plasma (NHP) was incubated with (+ CpaA) or without (− CpaA) culture supernatant from ATCC 17978/p*cpaAB*, diluted 1:100 and added to different factor deficient plasma as indicated. Clotting time was determined using an aPTT assay. ***, *P* ≤ 0.001 by ordinary one-way ANOVA; *n* = 6. (B) NHP was incubated with (+) or without (−) CpaA present in culture supernatant from ATCC 17978/p*cpaAB* for 20 min. fXII-deficient plasma incubated without CpaA was used as a control. Samples were subjected to SDS-PAGE and immunoblotting. fXII was detected using anti-fXII antibody. A representative blot is shown. Mw, molecular weight. (C) Purified human fXII was incubated with (+ CpaA) or without (− CpaA) culture supernatant from ATCC 17978/p*cpaAB*, followed by incubation without or with aPTT reagent to activate fXII and analyzed in a fluorogenic assay against the low-molecular-weight substrate z-GlyGlyArg-AMC to measure fXII proteolytic activity. ****, *P* ≤ 0.0001 by ordinary one-way ANOVA; *n* = 6.

Since the aPTT assay primarily measures the intrinsic coagulation pathway, to identify the target of CpaA, we used a modified aPTT factor assay with a variety of factor-deficient human plasma samples. Normal (healthy) plasma samples were first incubated with CpaA from culture supernatant of ATCC 17978/p*cpaAB* or LB medium as a control. The samples were then diluted 1:100 into various human plasma samples lacking specific factors of the intrinsic pathway, and an aPTT assay was performed. In this way, the factor inactivated by CpaA in the normal plasma will not be able to complement the specific factor-deficient plasma. The finding that the clotting time was increased only when CpaA-treated normal plasma was added to fXII-deficient plasma suggested that fXII is cleaved and inactivated by CpaA (Fig. 2A). These results were confirmed by immunoblotting (Fig. 2B), which showed that CpaA cleaves fXII in human plasma, and by analyzing the effect of CpaA on purified fXII in a fluorogenic assay against a low-molecular-weight substrate (Fig. 2C), showing that CpaA prevents the conversion of fXII into an active serine protease.

### Identification of cleavage sites in fXII.

**FIG 3:**
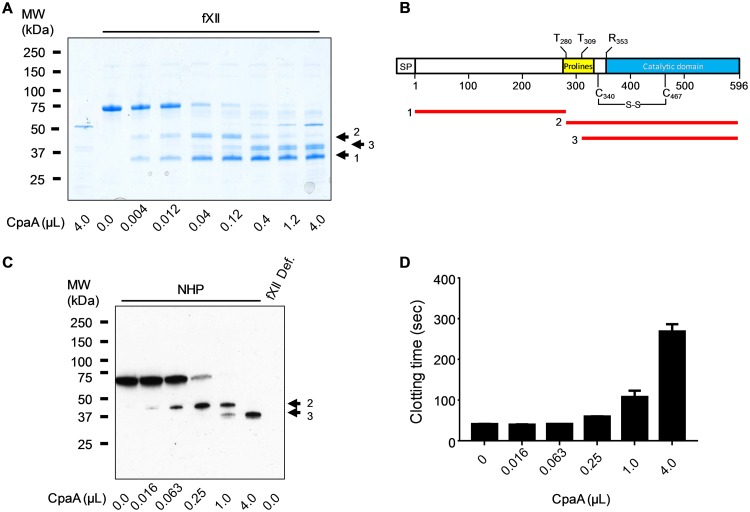
CpaA cleaves human factor XII twice. (A) Purified human fXII was incubated with increasing amounts of culture supernatant containing CpaA, followed by SDS-PAGE and Coomassie blue staining. CpaA and fXII alone are shown as controls (first and second lanes). The positions of the three fXII fragments generated following cleavage are indicated as arrows labeled 1, 2, and 3 on the right. A representative gel is shown. (B) Schematic representation of fXII and the three fXII fragments generated following cleavage by CpaA between Pro279Thr280 and Pro308Thr309. Signal peptide (SP), proline-rich domain (Prolines), and the catalytic domain containing the fXII activation site (Arg353Gln354) are indicated. (C and D) Increasing amounts of culture supernatant from ATCC 17978/p*cpaAB* were incubated with normal human plasma for 20 min. Samples were subjected to SDS-PAGE and immunoblotting, and fXII was detected using anti-fXII antibody. (C) A representative blot is shown. (D) The samples were also analyzed in an aPTT assay; *n* = 6.

Treatment of purified human fXII with increasing amounts of CpaA from culture supernatant of ATCC 17978/p*cpaAB* followed by SDS-PAGE analysis identified two distinct cleavage events that generated three fXII fragments (Fig. 3A), whereas control culture supernatant from ATCC 17978 with empty vector had no effect on fXII (Fig. S5). The fragments were blotted, excised, and subjected to N-terminal sequencing by Edman degradation to identify the cleavage sites. The low-molecular-weight fragment 1 sequence indicated that it represents the very N-terminal portion of mature fXII starting with residue Ile20 (residue 1 of mature fXII). Fragment 3, which is a cleavage product of fragment 2, ran as a doublet on SDS-PAGE (Fig. 3A). Both bands of the doublet were found to possess identical N terminus, XXRTPPQSQX. The first two residues and the last residue could not be identified as they are O-linked glycosylated threonines (UniProtKB-P00748 and reference 27). However, as residues 3 through 9 were clearly identified for both fragments, the results suggest that the sequence represents residues 309 to 318 in the glycosylated proline-rich domain of fXII and that fragment 3 is generated by cleavage between Pro308 and Thr309 (Fig. 3B). The reason fragment 3 ran as a doublet with identical N termini is likely due to glycosylation heterogeneity as mass analysis of purified, intact fXII identified three major species with mass differences that were multiples of 657 Da, which is the mass of NeuAc-Hex-HexNAc modification (Fig. S6).

10.1128/mBio.01606-18.5 FIG S5 No cleavage of purified human fXII by the reference strain ATCC 17978. Purified human fXII was incubated with increasing amounts of culture supernatant from ATCC 17978 with empty vector pMMB67 (p) followed by SDS-PAGE and Coomassie staining. The culture supernatant (lane 1) and fXII alone (lane 2) are shown as controls. Download FIG S5, PDF file, 0.02 MB. Copyright © 2018 Waack et al. 2018 Waack et al. This content is distributed under the terms of the Creative Commons Attribution 4.0 International license.

10.1128/mBio.01606-18.6 FIG S6 Purified human fXII is heterogeneous. Purified fXII was subjected to LC-MS analysis on a Waters Qtof Premier mass spectrometer. A representative MS spectrum shows a size difference of 657 Da between the three major peaks, which is indicative of differences in NeuAc-Hex-HexNAc modification. Download FIG S6, PDF file, 0.01 MB. Copyright © 2018 Waack et al. 2018 Waack et al. This content is distributed under the terms of the Creative Commons Attribution 4.0 International license.

The results from the N-terminal sequencing of fragment 2 (indicated in Fig. 3A) were not interpretable, possibly due to the presence of several prolines and glycosylated residues. Therefore, we subjected this fragment, as well as fragment 1, to in-gel trypsin digestion and liquid chromatography followed by tandem mass spectrometry (LC/MS/MS). Identification of glycosylated and nonglycosylated peptides using Bionic and Mascot software and peptide coverage analysis indicated that fragment 1 spans residues 1 to 279 and fragment 2 consists of residues 280 to 610 of fXII (Table 1). These results suggest that CpaA cleaves between Pro279 and Thr280 in the glycosylated proline-rich region of fXII (Fig. 3B). Taken together with the finding that the second site of cleavage also occurs between a proline and threonine, these results suggest that CpaA has a preference for Pro-Thr peptide bonds.

**TABLE 1:** Peptide coverage of fXII fragments 1 and 2

fXII fragment	Spectral counts for:	
	Residues 1 to 279	Residues 280 to 610
1	560	43
2	22	700

When normal (healthy) human plasma (NHP) was incubated with increasing amounts of CpaA, followed by SDS-PAGE and immunoblot analysis, plasma fXII was similarly cleaved at two sites by CpaA (Fig. 3C; note that only full-length fXII and fragments 2 and 3 were detected by the monoclonal anti-fXII, suggesting that the antibody epitope is absent in fragment 1). Furthermore, when these CpaA-treated plasma samples were analyzed in the aPTT assay, the results indicated that inactivation of fXII by CpaA occurs only upon cleavage at the second site, Pro308-Thr309, of fXII (note that the increase in clotting time seen in Fig. 3D correlates with the appearance of fragment 3 in Fig. 3C).

### Specificity of CpaA.

**FIG 4:**
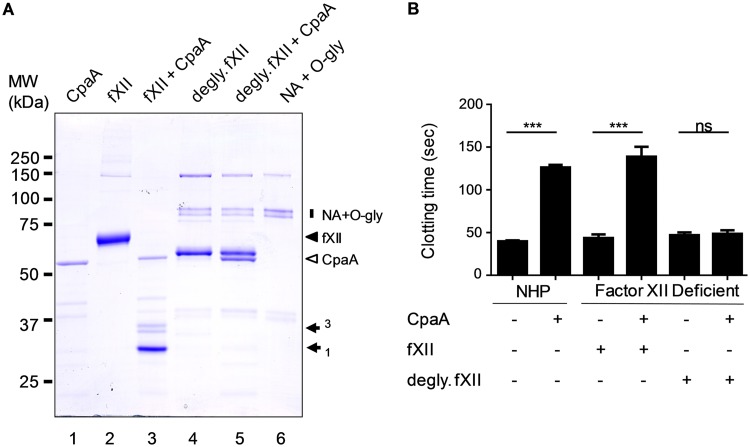
Glycosylation is necessary for cleavage of fXII by CpaA. (A) Purified fXII was incubated with and without neuraminidase and O-glycosidase and then treated with culture supernatant containing CpaA, followed by SDS-PAGE and Coomassie staining. Neuraminidase (NA) and O-glycosidase (O-gly) alone are shown as controls (lane 6; vertical bar on the right). The positions of fXII (filled arrowhead), CpaA (open arrowhead), and cleaved fXII fragments 1 and 3 (filled arrows) are indicated on the right. Note that fragment 3 runs as a doublet due to glycosylation heterogeneity. A representative gel is shown. (B) Purified fXII or deglycosylated fXII was added to fXII-deficient plasma following incubation with and without CpaA and tested for clotting in the aPTT assay. Results with normal human plasma (NHP) are shown for comparison. ***, *P* ≤ 0.001 by ordinary one-way ANOVA; *n* = 6. ns, not significant.

Both residues Thr280 and Thr309 in human fXII are modified with O-linked glycans (Swiss-Prot entry P00748 and references 27 and 28), suggesting that CpaA may have a preference for O-linked glycosylated residues. To determine whether glycosylation is required for CpaA cleavage, we treated fXII with neuraminidase and O-glycosidase to deglycosylate fXII before incubating it with CpaA. SDS-PAGE analysis indicated that deglycosylation resulted in increased mobility of fXII (Fig. 4A, compare lanes 2 and 4), but more importantly, prevented cleavage by CpaA (Fig. 4A, lane 5). The function of deglycosylated fXII, with and without treatment with CpaA, was also analyzed in a modified aPTT assay in which we determined the ability of purified fXII to restore normal clotting to fXII-deficient plasma. The results in Fig. 4B show that deglycosylated fXII fully restored clotting when added to fXII-deficient plasma, whether it had been incubated with CpaA or not, providing further support that deglycosylated fXII is resistant to CpaA.

**FIG 5:**
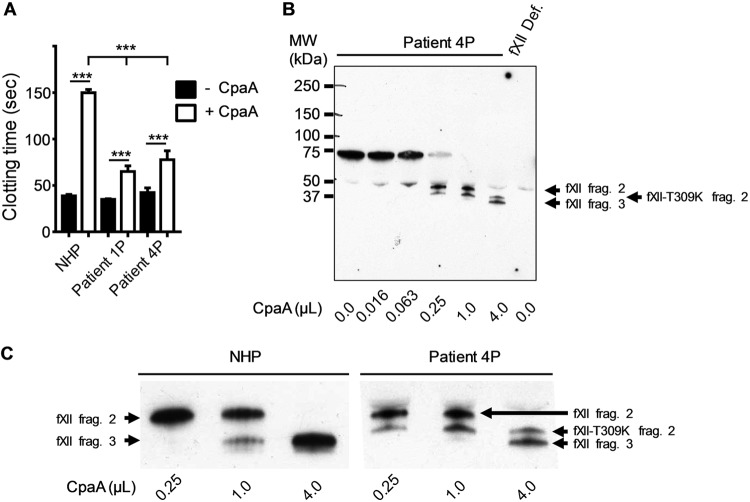
FXII-T309K is less sensitive to inactivation by CpaA than normal fXII. (A) Normal human plasma (NHP) and plasma samples from two patients with the fXII-T309K substitution (patient 1P and patient 4P) were incubated with (+ CpaA) or without (− CpaA) culture supernatant from ATCC 17978/p*cpaAB*, and an aPTT assay was performed. ***, *P* ≤ 0.001 by ordinary one-way ANOVA; *n* = 6 for NHP and *n* = 4 for the patient plasma samples. (B) Plasma from patient 4 was incubated with increasing amounts of culture supernatant from ATCC 17978/p*cpaAB*. fXII-deficient plasma incubated without CpaA was included as a control. Samples were subjected to SDS-PAGE and immunoblotting, and fXII was detected using anti-fXII antibody. (C) Enlargement of the region of the blot showing fragments 2 and 3 in panel B and shown next to the same region of the blot of NHP shown in Fig. 3C.

Interestingly, mutations in the CpaA-sensitive region of fXII have been identified in patients with hereditary angioedema type III (HAEIII) including two missense mutations at Thr309 and deletion of residues Lys305-Ala321 (29). Inheritance of either Lys or Arg substitutions at Thr309 (thus removing the O-linked glycosylation site) or the deletion are associated with an autosomal dominant form of this disorder in multiple families and leads to increased contact-mediated autoactivation of fXII that results in episodes of swelling of skin, life-threatening upper airway obstruction, and/or abdominal pain (28). When normal plasma (plasma from healthy individuals) and plasma samples from two different patients heterozygous for the fXII-Thr309Lys substitution were incubated with CpaA and then assayed for clotting in the aPTT assay, there was a significant increase in clotting time for normal plasma samples compared to patient plasma samples, suggesting that fXII-Thr309Lys is resistant to inactivation by CpaA (Fig. 5A). In subsequent experiments, we compared cleavage of fXII-Thr309Lys and fXII in patient plasma. While fXII-Thr309Lys was detected with reduced efficiency, the results indicated that both normal fXII and fXII-Thr309Lys in the patient plasma are cleaved by CpaA at the first site to generate fXII fragment 2 and fXII-Thr309Lys fragment 2 (Fig. 5B; note the mutant fragment demonstrates increased mobility due to the nonglycosylated substitution at position 309). As was shown above, normal fXII is then cleaved a second time to generate fXII fragment 3. However, the fXII-Thr309Lys fragment 2 remains uncleaved following incubation with CpaA (Fig. 5B and C), which is consistent with our finding that the clotting time of patient plasma, which has approximately 50% wild-type fXII and 50% mutant fXII, is only partially extended by CpaA compared to NHP (Fig. 5A).

### Other CpaA targets.

FXII is not the only target of CpaA. Even though we did not observe an effect in the PT assay (Fig. S4A), which suggests that fV was neither activated nor inactivated by CpaA, fV is cleaved by CpaA (Fig. S4B). Perhaps there are common O-linked glycosylation recognition sites in fXII and fV, but only cleavage of fXII leads to inactivation. Tilley et al. suggested that fibrinogen may also be a target of CpaA (17). However, we did not observe cleavage of fibrinogen in human plasma (Fig. S7).

10.1128/mBio.01606-18.7 FIG S7 CpaA does not cleave fibrinogen in human plasma. Normal human plasma (NHP) was incubated with or without CpaA, subjected to SDS-PAGE, and immunoblotted using anti-human fibrinogen antibodies. Purified human fibrinogen was used as control. A representative blot is shown. Download FIG S7, PDF file, 0.1 MB. Copyright © 2018 Waack et al. 2018 Waack et al. This content is distributed under the terms of the Creative Commons Attribution 4.0 International license.

### CpaA contributes to *in vivo* fitness in a bacteremia model.

**FIG 6:**
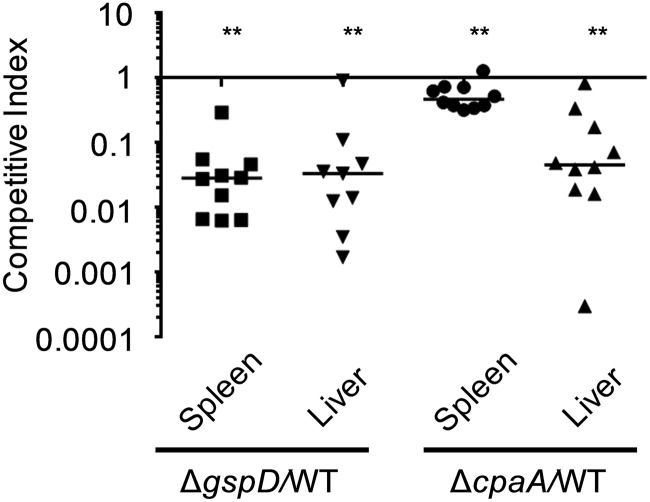
The T2SS and CpaA convey competitive advantage during colonization in a murine bacteremia model. Mice were coinoculated with equal amounts of either A. baumannii AB031 and AB031 Δ*gspD* or AB031 and AB031 Δ*cpaA*. After 24 h, the mice were euthanized, the spleen and livers were harvested, and the number of CFU was determined. The competitive index was calculated as follows: [output mutant CFU/output WT CFU]/[input mutant CFU/input WT CFU]. Bars indicate the median competitive index for each organ. Each symbol represents the competitive index for one mouse. **, *P* ≤ 0.01 by Wilcoxon signed rank test. Only nine data points are shown for the AB031 Δ*gspD/*WT AB031 liver samples due to one mouse sample having too many CFU to count.

We have previously shown that the T2SS supports survival of the A. baumannii reference strain ATCC 17978 in a bacteremia model of leukopenic CBA/J mice (8). Here, we determined whether the T2SS also promotes the *in vivo* survival of the *cpaA*-positive A. baumannii strain AB031, which was recently isolated from the bloodstream of a 55-year-old female patient (30). We pooled WT AB031 and the kanamycin-marked Δ*gspD* mutant cells at a ratio of 1:1 and inoculated CBA/J mice with 10^7^ colony-forming units (CFU) intravenously via the tail vein. Twenty-four hours later, the mice were euthanized, and spleens and livers were harvested, homogenized, and plated. In contrast to ATCC 17978 (4), AB031 did not require immunosuppression of mice for infection, and inoculation of 10^7^ bacteria resulted in 10^5^ CFU/liver/mouse 24 h postinoculation. The CFU of kanamycin-sensitive (WT AB031) and resistant (Δ*gspD* mutant) bacteria were counted, and competitive indices (CI) were calculated. With competitive indices significantly below 1, the mutant was outcompeted by the WT strain in both liver (CI = 0.033) and spleen (CI = 0.028), indicating that the T2SS contributes to *in vivo* fitness of AB031 (Fig. 6). Next we evaluated the role of CpaA in *in vivo* survival by analyzing the colonization of the gentamicin-marked Δ*cpaA* deletion mutant (17). The Δ*cpaA* deletion mutant was outcompeted by the WT strain in the liver (CI = 0.045) and to a lesser extent in the spleen (CI = 0.47) (Fig. 6), suggesting that CpaA is expressed and secreted *in vivo* and that it supports the survival of A. baumannii AB031 in the murine host. These results are consistent with the finding that dissemination of an A. nosocomialis *cpaA* mutant from the lungs in a pneumonia model of mice is reduced compared to WT A. nosocomialis when inoculated separately (16) and suggests that CpaA is an important Acinetobacter *in vivo* fitness factor.

### CpaA cleaves and inactivates murine fXII with reduced efficiency.

**FIG 7:**
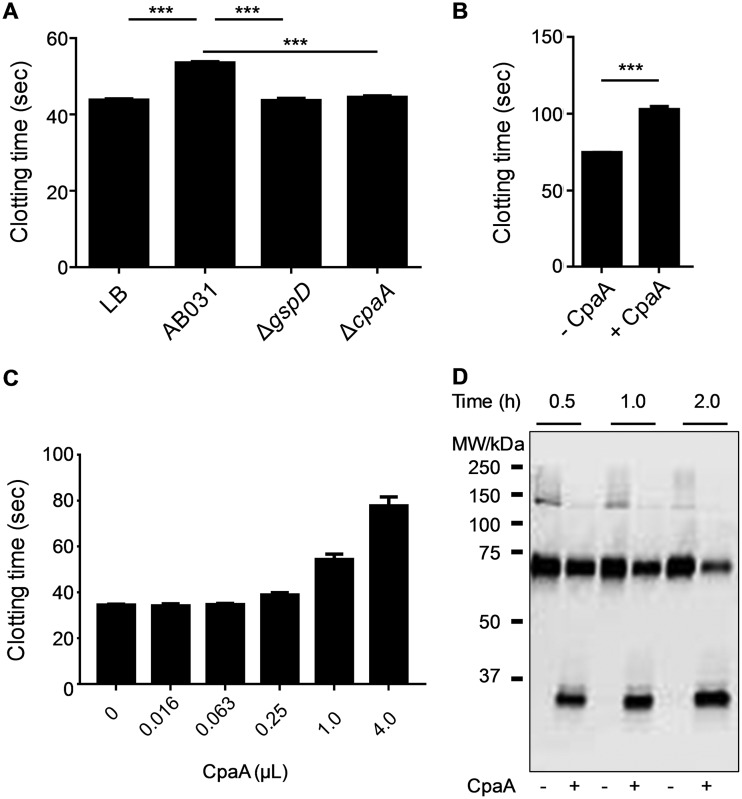
CpaA cleaves murine fXII with reduced efficiency. (A) aPTT after incubation of murine plasma with culture supernatants from strain AB031, AB031 Δ*cpaA*, or AB031 Δ*gspD*. The growth medium LB served as a negative control. ***, *P* ≤ 0.001 by ordinary one-way ANOVA; *n* = 6. (B) Murine plasma incubated with or without CpaA, diluted 1:100, added to human fXII-deficient plasma and tested in an aPTT assay. ***, *P* ≤ 0.001 by ordinary one-way ANOVA; *n* = 6. (C) Increasing amounts of culture supernatant from ATCC 17978/p*cpaAB* were incubated with mouse plasma for 20 min, and an aPTT assay was performed; *n* = 6. (D) Mouse plasma was incubated without (−) or with (+) 14 µl culture supernatant from ATCC 17978/p*cpaAB* for 0.5, 1.0, and 2.0 h. Mouse fXII was detected using an anti-fXII antibody. A representative blot is shown.

CpaA produced and secreted by the WT A. baumannii AB031 strain also interferes with clotting of murine plasma, although the efficiency in murine plasma appears to be reduced compared to human plasma (Fig. 7A). A factor assay similar to the one used in Fig. 2 was performed but with CpaA-treated normal mouse plasma used to reconstitute factor XII-deficient human plasma confirmed that fXII is also the target of CpaA in mouse plasma (Fig. 7B). To further investigate the reduced effect of CpaA on clotting in murine plasma, increasing amounts of CpaA were incubated with murine plasma, followed by aPTT analysis (Fig. 7C). While incubation of 1 µl and 4 µl of CpaA with human plasma for 20 min extended the clotting time from 40 s to 107 and 269 s (Fig. 3D), respectively, it only increased the clotting time of mouse plasma from 34 s to 54 and 78 s, respectively (Fig. 7C). Even when the amount of CpaA and the incubation time of murine plasma with CpaA was increased to 14 µl and 2 h, respectively, murine fXII was not fully cleaved by CpaA (Fig. 7D). These findings indicate that murine fXII is less sensitive to CpaA cleavage than human fXII.

### CpaA cleaves human fXII expressed in mice.

**FIG 8:**
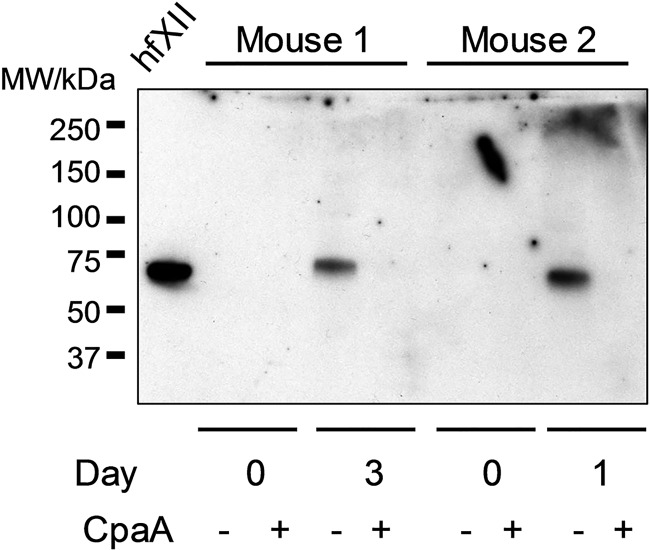
Human fXII expressed in mice is sensitive to CpaA. Plasma from two different mice injected hydrodynamically with pLIVE expressing human fXII cDNA were collected on day 3 (mouse 1) and day 1 (mouse 2) and incubated without (−) or with (+) CpaA-containing culture supernatants from ATCC 17978/p*cpaAB*. Control plasma samples were collected from each mouse prior to plasmid injection (day 0) and used as a negative control. Purified human fXII (hfXII; 10 ng) is included as a positive control on the left. A representative blot is shown.

**FIG 9:**
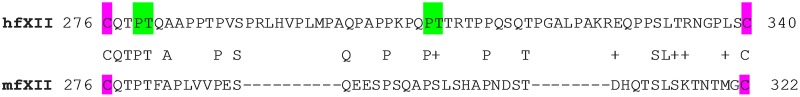
Low sequence conservation between the proline-rich domain of human and mouse fXII. The primary sequence, including the N-terminal signal peptide of human fXII (hfXII) was aligned with that of mouse fXII (mfXII). The sequence between the conserved cysteines at positions 276 and 340 (highlighted in magenta) containing the proline-rich domain of hfXII is shown on the top. Note that this region is 18 residues shorter in mfXII (bottom). The sites cleaved by CpaA in hfXII are marked in green.

To determine whether differences in the primary sequences of human and mouse fXII or whether other differences between human and mouse plasma are responsible for the difference in susceptibility to CpaA, we transiently expressed human fXII in mice and tested it for cleavage by CpaA. The cDNA for human fXII was cloned into pLIVE, an *in vivo* expression vector that allowed for expression of human fXII from the mouse albumin promoter in the liver following hydrodynamic tail vein injection of the plasmid DNA. Plasma samples from two different mice were obtained before (day 0) and after DNA injection (either day 1 or day 3), and the human fXII was tested for sensitivity to CpaA cleavage. Samples were subjected to SDS-PAGE and immunoblotted with sheep anti-human fXII antibodies that do not cross-react with mouse fXII (see day 0 samples in Fig. 8). Using the same amount of CpaA previously used for cleavage of fXII in human plasma and incubating for 20 min, we found that human fXII expressed in mice was fully sensitive to CpaA, suggesting that the difference in CpaA sensitivity between human and mouse fXII are likely due to differences in their primary amino acid sequences (Fig. 9).

## DISCUSSION

Our studies focused on the putative function of the metalloprotease CpaA, which is secreted by the T2SS in A. baumannii. Coagulation factor fXII was recognized as a substrate for CpaA in both human and murine plasma. N-terminal sequencing and mass spectrometry of human fXII fragments generated following treatment with CpaA localized the cleavage sites to Pro279Thr280 and Pro308Thr309 located in the proline-rich domain of fXII. These findings suggest that CpaA has specificity for Pro and Thr at the P1 and P1’ positions, respectively. Interestingly, both Thr280 and Thr309 are glycosylated residues, and deglycosylation of fXII prevents cleavage at both sites. Similarly, substitution of the glycosylated Thr309 with lysine protects fXII-Thr309Lys in plasma from patients with HAEIII from CpaA-mediated inactivation. If CpaA contributes to human disease, individuals with this mutation as well as the Thr309Arg substitution and the K305-A321 deletion may be partially protected from the effect of CpaA during A. baumannii infection (28, 29, 31, 32).

Glycosylation generally protects proteins from proteolysis; however, in the case of fXII, glycosylation makes it a target for proteolysis, as deglycosylation of fXII prevents cleavage. This finding adds CpaA to a small, but growing list of bacterial proteases with specificity toward glycosylated residues. For example, IgA proteases produced by pathogens such as Streptococcus pneumoniae, Haemophilus influenzae, and *Neisseria* species specifically target proline-threonine and proline-serine peptide bonds in the glycosylated hinge region of human IgA1 (33). Another example, StcE from enterohemorrhagic Escherichia coli cleaves the glycosylated N-terminal domain of C1-esterase inhibitor (34).

The primary amino acid sequences of human and mouse fXII are 71% identical; however, the glycosylated proline-rich domain greatly differs between the two fXII species. Between Cys276 and Cys340 in human fXII, there is no significant homology observed with mouse fXII and the mouse sequence is 18 residues shorter (Fig. 9). While mouse fXII does have sites equivalent to Pro279Thr280 and Pro308Thr309, other differences in the proline-rich domain may be responsible for the reduced cleavage efficiency of mouse fXII by CpaA.

Our previous work demonstrated the advantage of possessing the T2SS for colonization of ATCC 17978 (8). We now show that this is also true for the AB031 strain. However, in contrast to ATCC 17978, neutropenia is not required for dissemination and colonization of AB031 in the liver and spleen, suggesting that AB031 is better able to resist host defense mechanisms. The importance of the T2SS in multiple strains of A. baumannii as well as A. nosocomialis for increased survival *in vivo* is indicative of a widespread role of the system and its substrates in the species as a whole. While the defect in colonization was equivalent for the spleen and liver for the T2SS mutant, there was a difference between the organs for the Δ*cpaA* mutant with the liver showing a greater difference than the spleen. This requires further investigation; however, it is interesting to note that the liver is the primary site of fXII synthesis (35), and it is possible that A. baumannii is exposed to higher levels of fXII in this organ, thus decreasing the competitiveness of the Δ*cpaA* mutant. An alternative explanation is that the WT and mutant strains occupy a much smaller space in the spleen and the mutant is complemented in *trans* with secreted CpaA from neighboring WT bacteria. However, the latter scenario is less likely, as a similar competition assay for WT and a Δ*lipaA* mutant to determine the contribution of a secreted lipase to *in vivo* fitness did not indicate a reduced requirement for lipase in the spleen (8).

The role of fXII is not yet fully understood, since while fXII deficiency demonstrates a significant defect in the aPTT assay of coagulation, it is not associated with bleeding (36). However, recent studies have shown that fXII promotes intravascular thrombus formation (21, 37, 38). CpaA may inactivate fXII to prevent the formation of intravascular clots that would otherwise trap A. baumannii during bacteremia. Without the hindrance of a clot, A. baumannii may disseminate and gain access to other organs. Another example of a pathogen that is hypothesized to evade clot-mediated immobilization is group A streptococcus (GAS). GAS produces streptokinase, which can degrade the major constituent of the clot, fibrin, through conversion of plasminogen to plasmin, thus enabling GAS to escape and disseminate (39). Cleavage of fXII by CpaA may also affect the kallikrein/kininogen pathway. fXIIa is the primary activator of prekallikrein to kallikrein which, in turn, liberates bradykinin from high-molecular-weight kininogen, and in mice deficient in fXII, bradykinin levels are significantly reduced (40). Bradykinin is a peptide hormone with a wide array of roles. It promotes efflux of immune cells by increasing vascular permeability and vasodilation by binding to B-2 receptors (41). Bradykinin, in its metabolized form, binds B-1 receptors (41). Engagement of both the B-2 and B-1 receptors leads to release of immune and inflammation mediators that are immune cell dependent (42). By inactivating fXII, CpaA may reduce the generation of bradykinin, potentially reducing the recruitment of immune mediators, including neutrophils, and thus support survival of A. baumannii. In a very recently reported study of a sterile inflammation model, a direct role for the zymogen form of fXII in neutrophil trafficking was identified that is independent of fXIIa protease function or the kallikrein/kininogen pathway (22). Cleavage of fXII by CpaA could therefore affect the upregulation of neutrophil functions and prevent important processes such as chemotaxis and formation of neutrophil extracellular traps, which are critical for innate antibacterial processes. Finally, fXII is also associated with the classical pathway of the complement system as active fXII is capable of activating C1 esterase, and therefore, CpaA-mediated inactivation of fXII could also interfere with a branch of the acquired immune system.

## MATERIALS AND METHODS

### Bacterial strains and growth conditions.

Acinetobacter baumannii ATCC 17978 and AB031 and their isogenic mutants were cultured in Luria-Bertani (LB) broth at 37°C. Carbenicillin (100 µg/ml) or kanamycin (50 µg/ml) was used for plasmid maintenance. The bacterial strains and plasmids used in this study are given in Table 2.

**TABLE 2:** Plasmids and strains used in this study

Plasmid or strain	Relevant characteristic(s)	Reference or source
Plasmids		
pK18mobsacB	Suicide vector containing *sacB* (Km^r^)	44
pCVD442	Suicide vector containing *sacB* (Ap^r^)	45
pMMB67EH	Low-copy, IPTG-inducible vector (Ap^r^)	46
pMMB960	pMMB67EH with substitution of Ap^r^ for Kan^r^	M. Bagdasarian, Michigan State University
p*cpaAB*	pMMB67EH-*cpaAB*	This study
p*cpaA*-*kan*	pMMB960-*cpaA*	This study
pcpaAB-*kan*	pMMB960-*cpaAB*	This study
p*gspD*	pMMB67EH-*gspD*	8
pLIVE	Used for gene delivery and expression in mouse liver	Mirus Bio LLC
pLIVE-hfXII	pLIVE with cloned human fXII cDNA	This study

*E. coli* strains		
MC1061	F^−^ *lac* mutant; K-12 laboratory strain	47
MM294/pRK2013	Helper strain for conjugation	48
Y327λpir MC1061	λpir lysogen; permits replication of pCVD442F^−^ *lac* mutant; K-12 laboratory strain	49

*A. baumannii* strains		
AB031	Clinical isolate	17
AB031Δ*cpaA*	Replacement of *cpaA* with *aacC1* (Gm^r^)	17
AB031Δ*gspD*	Replacement of *gspD* with *aph-3* (Km^r^)	This study
ATCC 17978	Wild type for T2SS	ATCC
ATCC 17978Δ*gspD*	Replacement of *gspD* with *aph-3* (Km^r^)	8

### Construction of Δ*gspD* strains.

Chromosomal DNA isolated from the AB031 strain was used as the template for PCR. PCRs were carried out with Phusion DNA polymerase (Thermo Fisher). Primers were synthesized by IDT Technologies.

To generate the AB031Δ*gspD* strain, we used a previous plasmid construct, pCVDΔ*gspD* (8). This construct was conjugated from the Escherichia coli strain SY327λpir into the A. baumannii AB031 strain. Transconjugates in which pCVDΔ*gspD* had recombined into the A. baumannii genome were selected on LB agar containing carbenicillin. To select for the second recombination event, individual colonies were cultured overnight in LB broth, diluted, cultured to late log phase, and spread on LB agar containing 3% sucrose. Sucrose- and kanamycin-resistant and carbenicillin-sensitive isolates were considered positive for the recombination event.

### Construction of p*cpaA* and p*cpaAB* plasmids.

The *cpaA* and *cpaB* genes were amplified from chromosomal AB031 DNA using primers 5′-GAGGAGCTCTGGTTTGCTAACCTGC-3′ and 5′-GAGGCATGC TCTCTACCAGAACCGTT-3′. The 2,532-bp product was digested with SacI and SphI and ligated into a low-copy-number, broad-host vector pMMB67EH to make p*cpaAB*. The construct was verified by sequencing and conjugated from the E. coli strain MC1061 into WT and Δ*gspD* A. baumannii 17978 strains to make WTp*cpaAB* and Δ*gspD*p*cpaAB*.

### *In vivo* competition assay.

Competition assays were carried out as described previously (8). Briefly, overnight cultures of strains AB031 and AB031Δ*gspD* or AB031Δ*cpaA* were suspended in phosphate-buffered saline (PBS). Inocula of 10^7^ cells of equal amounts of AB031 and AB031Δ*gspD* were coadministered via tail vein injection into 8-week-old female CBA/J mice (Jackson Laboratory). Spleens and livers were removed after mice were euthanized 24 h postinoculation. Organs were homogenized in PBS and plated onto LB agar with and without kanamycin. CFU counts were determined and used to calculate the competitive index (CI) as follows: CI = ([mutant CFU/WT CFU]/[mutant input CFU/WT input CFU]).

### Concentration of CpaA in supernatant.

Cultures (10 ml) of each A. baumannii strain were grown for 16 h in LB. The supernatant was separated from the cells by centrifugation at 1,250 × *g* for 10 min and filter sterilized. The supernatants were concentrated 100× using Amicon Ultra Centrifugal filters with a 30-kDa cutoff. To determine the approximate concentration of CpaA present in concentrated supernatant of 17978/p*cpaAB* cultures, the supernatant was subjected to SDS-PAGE and Coomassie blue staining and the amount of CpaA was compared to known amounts of BSA, a protein of similar size.

### Activated partial thromboplastin time (aPTT) assay.

Concentrated cell-free supernatants were mixed with normal (healthy) human pooled plasma samples (George King) for 20 min at 37°C. An equal volume of aPTT reagent (Pacific Hemostasis) was added. After incubation for 5 min, CaCl_2_ (final 10 mM) was added. Clot times were determined using an Amelung KC4A Micro Coagulation analyzer.

To identify the target of CpaA, a modified aPTT assay was performed by first incubating concentrated AB031 supernatant with normal plasma. After incubation, the plasma was diluted 1:100 and added to human plasma samples derived from donors deficient in congenital coagulation factor (George King), and an aPTT assay was performed as described above.

### Prothrombin time (PT) assay.

Concentrated cell-free supernatant was incubated with normal human plasma for 20 min at 37°C. After incubation, PT reagent was added, and clot times were determined using an Amelung KC4A Micro Coagulation analyzer.

### fXII proteolytic activity assay.

Commercially available human fXII was incubated with or without concentrated cell-free supernatant containing CpaA at 37°C. These samples were then incubated with or without aPTT reagent, and the proteolytic activity of fXIIa was measured kinetically against z-GlyGlyArg-AMC for 10 min at 37°C using excitation and emission wavelengths of 370 nm and 440 nm, respectively.

### SDS-PAGE and immunoblot analysis.

Samples were boiled in SDS sample buffer, subjected to SDS-PAGE on 4% to 12% Bis-Tris polyacrylamide gels (NuPAGE; Invitrogen), and visualized using a Typhoon Trio variable mode imager system and ImageQuant software. CpaA was identified by liquid chromatography-mass spectrometry (LC-MS/MS) of gel-excised material containing A. baumannii culture supernatant (MS Bioworks, Ann Arbor, MI).

Samples were subjected to SDS-PAGE and immunoblotting using anti-human fXII (Molecular Innovations), 1:5,000 sheep anti-human fV (Molecular Innovations), and 1:5,000 goat anti-human fibrinogen (Sigma). Horseradish peroxidase-conjugated secondary antibodies (fXII [1:2,000], fV 1:10,000], and fibrinogen [1:1,000]; Jackson Laboratories) were used, and the blots were visualized with SuperSignal West Pico Substrate (Thermo Scientific). Developed blots were imaged using a Typhoon Trio imager (GE Healthcare). Rabbit anti-mouse fXII (Molecular Innovations) and Alexa Fluor 680-conjugated goat anti-rabbit antibodies were used for detection of mouse fXII. Blots were imaged by using the Odyssey imaging system.

### N-terminal sequencing.

Purified fXII was treated with CpaA-containing culture supernatants, subjected to SDS-PAGE, and transferred to PVDF membrane using 10 mM 3-[cyclohexylamino]-1-propanesulfonic acid (CAPS), 10% methanol, pH 11. The membrane was stained with Coomassie brilliant blue R-250, destained, washed with water, and then air dried. The fXII fragments were cut out and subjected to automated N-terminal sequencing (Edman degradation) using the ABI 492 protein sequencer system (Protein Chemistry Laboratory at Texas A&M University). The sequencing results were confirmed with a second set of fXII cleavage products.

### Intact mass determination of fXII.

Commercially available human fXII (6 µg in 4 mM sodium acetate/0.15 M sodium chloride, pH 5.3) was subjected to LC-MS analysis on a Waters quadrupole time-of-flight (Qtof) Premier mass spectrometer in-line with a Waters NanoAcquity UPLC via a nanoelectrospray ion source. The sample was injected on a Poros R1 protein column using the 0.1% formic acid in water as mobile phase A and 0.1% formic acid in acetonitrile as mobile phase B with a flow rate of 10 μl per minute. The initial mobile phase composition was 1% mobile phase B, which was held for 2 min and then increased to 95% mobile phase B at 5 min to elute the protein. Mobile phase B was returned to 1% at 6 min and recycled to 95% mobile phase B at 7 min and then back to 1% mobile phase B at 8 min for the remainder of the 15-min total run time.

The raw data were processed in MassLynx v4.1 software. Raw data containing protein signal were combined and then smoothed using a Savitzky-Golay method with a window size of 3 (scans) and number of smooths equal to 2. Horse heart myoglobin protein from Sigma-Aldrich (catalog no. M1882) was analyzed at a concentration of 10 pmol/μl to obtain a correction factor that was applied to the fXII spectrum to obtain mass measurement accuracy. The fXII multiply charged spectrum was deconvoluted to M+H average mass using Waters MaxEnt1 software.

### GeLC-MS/MS analysis.

The Coomassie blue-stained bands containing fXII fragments 1 and 2 were excised and subjected to in-gel digestion with trypsin performed with a ProGest robot (DigiLab) by the following protocol: (i) washed with 25 mM ammonium bicarbonate and then with acetonitrile; (ii) reduced with 10 mM dithiothreitol at 60°C, followed by alkylation with 50 mM iodoacetamide at room temperature (RT); (iii) digested with trypsin (Promega) at 37°C for 4 h; and (iv) quenched with formic acid and analyzed without further processing. The digest was analyzed by nano LC-MS/MS with a Waters NanoAcquity HPLC system interfaced to a ThermoFisher Q Exactive. Peptides were loaded on a trapping column and eluted over a 75-µm analytical column at 350 nl/min; both columns were packed with Luna C18 resin (Phenomenex). The mass spectrometer was operated in data-dependent mode, with the Orbitrap operating at 70,000 FWHM and 17,500 FWHM for MS and MS/MS, respectively. The fifteen most abundant ions were selected for MS/MS. Data were searched using a local copy of Mascot (Matrix Science Ltd.) with the following parameters: Enzyme, Semi-Trypsin; database, Swiss-Prot Human (concatenated forward and reverse plus common contaminants); fixed modifications, Carbamidomethyl (C); variable modifications, Oxidation (M), Acetyl (N-term), Pyro-Glu (N-term Q), and Deamidation (N,Q); mass values, monoisotopic; peptide mass tolerance, 10 ppm; fragment mass tolerance, 0.02 Da; and maximum missed cleavages, 2. Mascot DAT files were parsed into Scaffold (Proteome Software Inc.) for validation and filtering and to create a nonredundant list per sample. Data were filtered using a 1% protein and peptide FDR and requiring at least two unique peptides per protein. The data were also searched using the Byonic software (Protein Metrics) to identify glycosylated peptides. For N-glycans, a database of 309 mammalian glycans was searched; for O-glycans, the database contained 70 human glycans. The peptide spectral counts for fXII fragments 1 and 2 listed in Table 1 were calculated from the Byonic data.

### Deglycosylation.

Purified fXII was treated with O-glycosylase (endo-α-*N*-acetylgalactosaminidase) and neuraminidase (acetyl-neuraminyl hydrolase) for 4 h at 37°C in Glycobuffer according to the manufacturer’s instructions (New England BioLabs) to remove O-linked disaccharide carbohydrates.

### Cloning and expression of human fXII in mice.

fXII was PCR amplified from the pcDNA5/FRT vector (without the His tag) and subcloned into pLIVE at the AscI and SgrAI restriction sites, fusing tandem, C-terminal E and FLAG tags to fXII. Expression is driven by a murine albumin promoter. Expression plasmids were delivered into 129S1/SvIm mice by hydrodynamic tail vein injection as previously described (43). Blood was collected from the retro-orbital plexus or the inferior vena cava and anticoagulated with sodium citrate at a 1:9 (vol/vol) ratio.

### Statistical analysis.

Ordinary one-way ANOVAs were performed on the aPPT and modified aPTT tests. A Wilcoxon signed rank test was used to analyze the *in vivo* murine data.

### Ethics statement.

All mouse care and procedures were performed according to the protocols (PRO00007111, PRO00005191, and PRO00007879) approved by the Institutional Animal Care and Use Committee at the University of Michigan. These protocols are in complete compliance with the guidelines for humane use and care of laboratory animals mandated by the National Institutes of Health.

### Patients.

The plasma samples from patients heterozygous for fXII-Thr309Lys were obtained with informed consent at Grenoble University Hospital Centre, Grenoble, France.
