# Supplementary material for: CpaA Is a Glycan-Specific Adamalysin-like Protease Secreted by Acinetobacter baumannii That Inactivates Coagulation Factor XII
Source: mBio. 2018 Dec 18;9(6):e01606-18. doi: 10.1128/mBio.01606-18 (PMC6299215; doi:10.1128/mBio.01606-18)
Supplement: FIG S2 [file mbo006184226sf2.pdf]

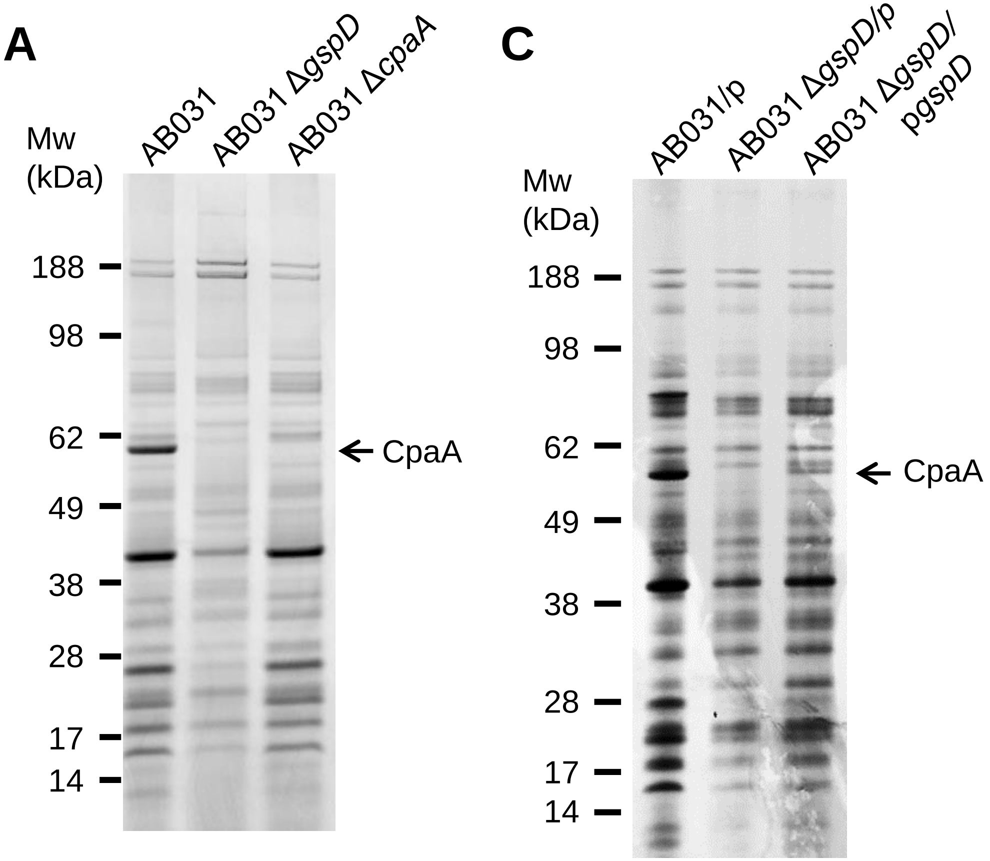

B

```
MNFKLKTSLIIGAIVASSLVYAATVLSPNQNNNSGSIPSGYSDLEFNLANGNWVKNLTLP
TSANNLDKITIRSSAAYSSYLDTSNTNIPLEVLKINSGDVYQFIFNSSQNKWIAQLATVS
PTNGATYEVVPLTTASMQKVLIQNEKWAQTIALPSDVRDGTTVQVVSTASASSDIDKTNL
LFPSSFILKNGSEYWFKYYSALGKWVPEYIKPQKLNVQQIGTSLATVNSPLTEVSFGDGN
WVSNFTLPTTASDRDKIIIKSTATWSAKINNTNINSQATLTLKTGDQYEFMYVSDKGYWQ
LISSPTKVIDSAATIPATLPNMTQPTLKVKLSTSNWQPTLQLPAKAQVGDKVVIVSNASA
DTYINAANGLSTAIKNGENRRFIYTAQGWTVDSYTIDMLLVSSPEVNSILGESAAKLRMI
EGVNLTNLTAENSNARFYLRNVGYLTYKIPAATLKEAISTGRDDTTVQNERKRVLADGVY
YQGNEPGDGGCGWAWINASAYNMIGANDIAGCSFAAMRHEVGHNLGLYHNGSTNIGSGFA
HPLGSTAMGGNNINFYSSPYLYNPKYGVRLGVEGKIDAVSVINLNAQKISLYN
```

Figure S2. Secretion of CpaA is dependent on the *A. baumannii* T2SS. A. Concentrated culture supernatants from AB031 and the T2SS mutant Δ*gspD* were analyzed by SDS-PAGE and Coomassie staining. The AB031 Δ*cpaA* mutant served as a negative control. **B.** CpaA was excised from the gel and subjected to LC-MS/MS analysis, which identified 45 exclusive unique peptides, 121 exclusive unique spectra and a total of 448 spectra. The resulting sequence coverage (highlighted in yellow) was 84%. No peptide representing the signal peptide was identified as the signal peptide is removed during transport of CpaA across the cytoplasmic membrane. **C.** Concentrated culture supernatants from AB031 and the T2SS mutant Δ*gspD* with empty vector pMMB67 (p) or plasmid-encoded *gspD* (p*gspD*) were analyzed by SDS-PAGE and Coomassie staining.
