# Supplementary material for: CpaA Is a Glycan-Specific Adamalysin-like Protease Secreted by Acinetobacter baumannii That Inactivates Coagulation Factor XII
Source: mBio. 2018 Dec 18;9(6):e01606-18. doi: 10.1128/mBio.01606-18 (PMC6299215; doi:10.1128/mBio.01606-18)
Supplement: FIG S3 [file mbo006184226sf3.pdf]

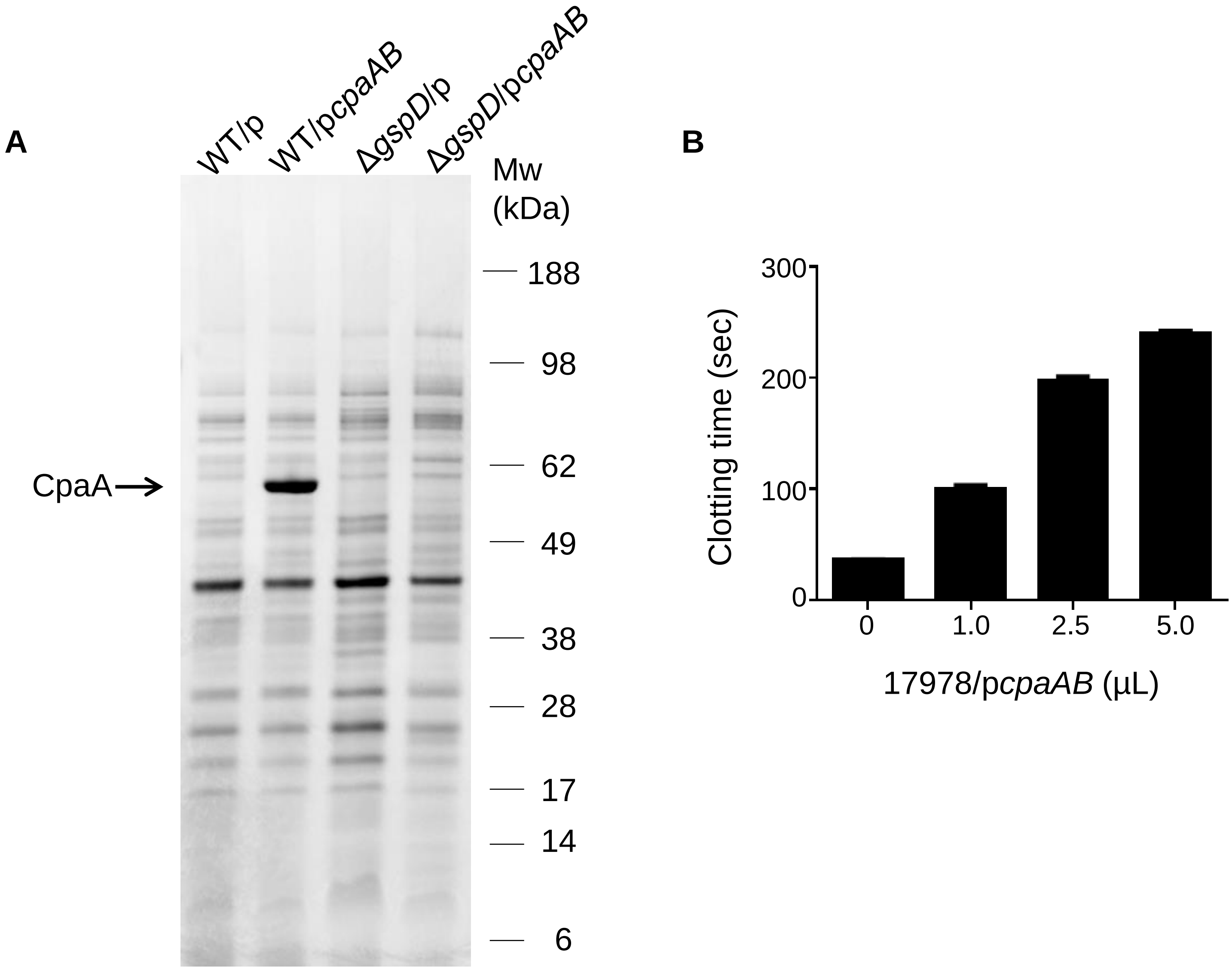

Figure S3. Overexpression. secretion and activity of CpaA in the ATCC 17978 strain. A. Concentrated culture supernatants from ATCC 17978 or the Δ*gspD* mutant containing empty vector (p) or a plasmid carrying the *cpaA* and *cpaB* genes (p*cpaAB*) were analyzed by SDS-PAGE and Coomassie staining. **B.** Increasing amounts of culture supernatant from 17978/p*cpaAB* were added to normal human plasma (NHP), and an aPTT assay was performed; n=6.
