# Supplementary material for: CpaA Is a Glycan-Specific Adamalysin-like Protease Secreted by Acinetobacter baumannii That Inactivates Coagulation Factor XII
Source: mBio. 2018 Dec 18;9(6):e01606-18. doi: 10.1128/mBio.01606-18 (PMC6299215; doi:10.1128/mBio.01606-18)
Supplement: FIG S4 [file mbo006184226sf4.pdf]

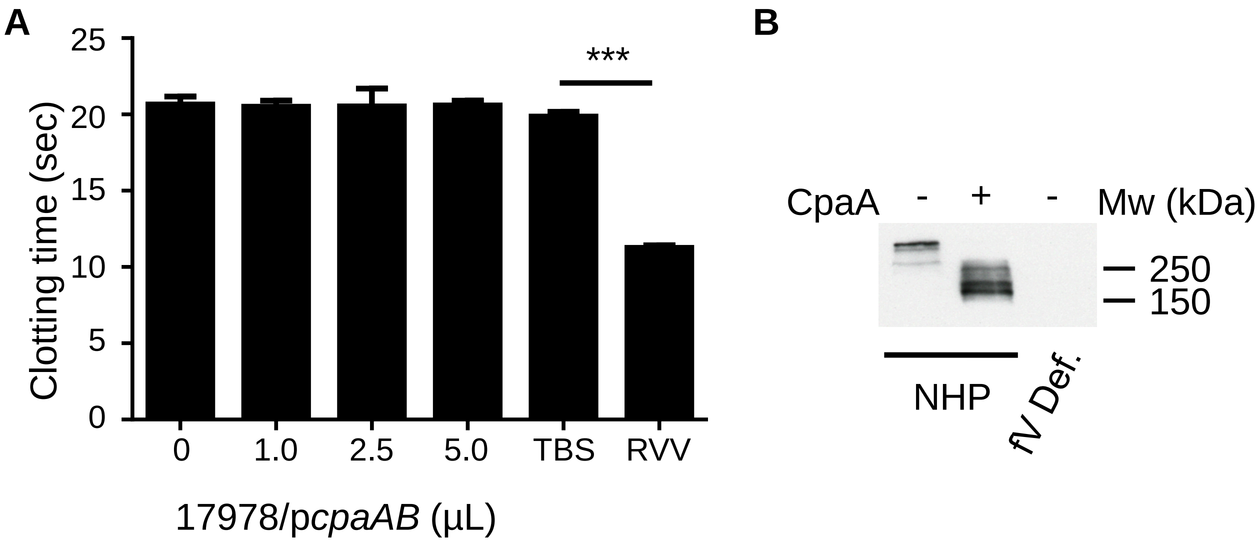

Figure S4. CpaA has no effect on clotting in a PT assay. A. Increasing amounts of supernatant from 17978/p*cpaAB* containing CpaA was added to NHP and analyzed for clotting using a PT assay; n=6, ***p<0.001. The snake venom protease RVV-V, a factor V activator, and the buffer TBS were included as controls. **B.** NHP was incubated with or without CpaA and subjected to SDS-PAGE and immunoblotting using α-fV antibody. fV deficient plasma incubated in the absence of CpaA was used as a negative control. Representative blot is shown.
