# Supplementary material for: CpaA Is a Glycan-Specific Adamalysin-like Protease Secreted by Acinetobacter baumannii That Inactivates Coagulation Factor XII
Source: mBio. 2018 Dec 18;9(6):e01606-18. doi: 10.1128/mBio.01606-18 (PMC6299215; doi:10.1128/mBio.01606-18)
Supplement: FIG S5 [file mbo006184226sf5.pdf]

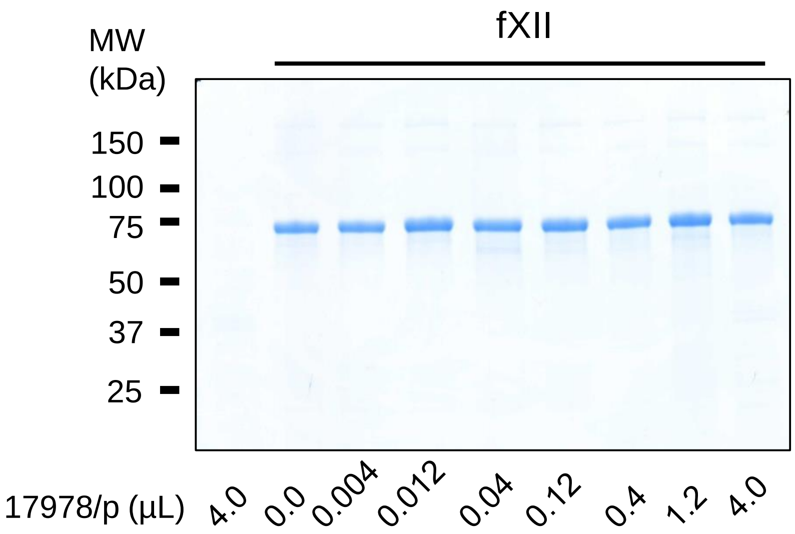

Figure S5. No cleavage of purified human fXII by the reference strain ATCC 17978. Purified human fXII was incubated with increasing amounts of culture supernatant from ATCC 17978 with empty vector pMMB67 (p) followed by SDS-PAGE and Coomassie staining. The culture supernatant (lane 1) and fXII alone (lane 2) are shown as controls.
