# Supplementary material for: CpaA Is a Glycan-Specific Adamalysin-like Protease Secreted by Acinetobacter baumannii That Inactivates Coagulation Factor XII
Source: mBio. 2018 Dec 18;9(6):e01606-18. doi: 10.1128/mBio.01606-18 (PMC6299215; doi:10.1128/mBio.01606-18)
Supplement: FIG S6 [file mbo006184226sf6.pdf]

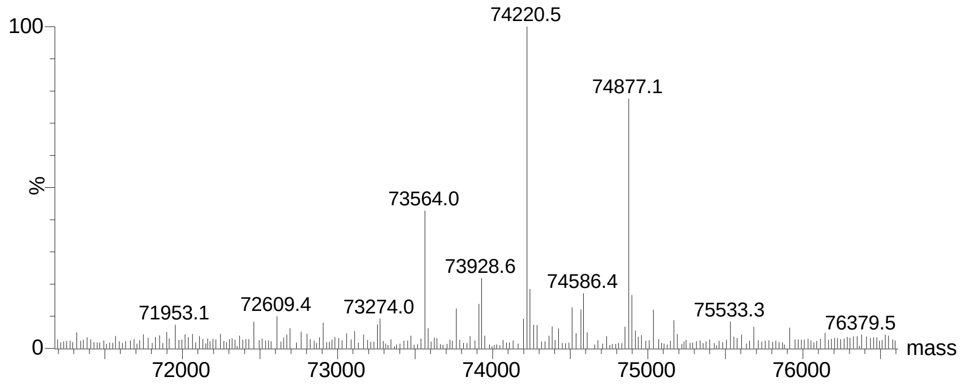

Figure S6. Purified human fXII is heterogeneous. Purified fXII was subjected to LC-MS analysis on a Waters Qtof Premier mass spectrometer. Representative MS spectrum shows a size difference of 657 Da between the three major peaks, which is indicative of differences in NeuAc-Hex-HexNAc modification.
