# Supplementary material for: CpaA Is a Glycan-Specific Adamalysin-like Protease Secreted by Acinetobacter baumannii That Inactivates Coagulation Factor XII
Source: mBio. 2018 Dec 18;9(6):e01606-18. doi: 10.1128/mBio.01606-18 (PMC6299215; doi:10.1128/mBio.01606-18)
Supplement: FIG S7 [file mbo006184226sf7.pdf]

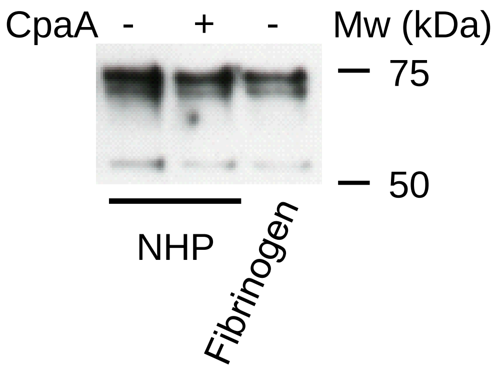

Figure S7. CpaA does not cleave fibrinogen in human plasma. Normal human plasma (NHP) was incubated with or without CpaA, subjected to SDS-PAGE, and immunoblotted using α-human fibrinogen antibodies. Purified human fibrinogen was used as control. Representative blot is shown.
